# 4339 Exploring Physician Investigator Clinical Trials Training and Quality Management Systems and its Implementation in Medical School Curriculums

**DOI:** 10.1017/cts.2020.129

**Published:** 2020-07-29

**Authors:** Sukhmani Kaur, Advaita Chandramohan, Eunjoo Pacifici

**Affiliations:** 1 University of Southern California

## Abstract

OBJECTIVES/GOALS: Although many physicians conduct clinical trials as Principle Investigators, a systematic training is often lacking. Instead, most receive on-site training, potentially compromising data quality and human subject safety. This research assesses the landscape for physician training through medical school curriculums. METHODS/STUDY POPULATION: This project explored training programs for physician researchers, specifically in the emerging field of quality management systems (QMS). To understand the scope of academic research available for QMS and Good Clinical Practice (GCP) training and lack of clinical trial training implemented in medical school curricula, a literature review was conducted. Available training for physicians was assessed through existing training programs from the FDA, NIH, DIAMOND, ACRP, and Google for accessibility in terms of costs, completion timelines and certification, format (online vs. in-person), and inclusion of GCP and QMS training in the curriculum RESULTS/ANTICIPATED RESULTS: Literature review revealed that not much is known about physician researcher training beyond the institutional requirement for minimal GCP review. Examination of select medical school curriculum also discovered a lack of clinical trial training for students interested in clinical research. Furthermore, existing training programs and modules available for physicians are limited as their syllabi do not include QMS training. In addition, these programs commonly have inaccessible registration links, are expensive, and have significant time commitments for in-person courses. These findings support the need for more accessible and effective training and certification tools for physician researchers. DISCUSSION/SIGNIFICANCE OF IMPACT : QMS training is not included medical school curricula or programs for physician researchers, potentially compromising data integrity and subject protection. This research supports the development of essential QMS training concepts and practical approaches for physician researcher clinical trials.

